# Manually curated and harmonised transcriptomics datasets of psoriasis and atopic dermatitis patients

**DOI:** 10.1038/s41597-020-00696-8

**Published:** 2020-10-13

**Authors:** Antonio Federico, Veera Hautanen, Nils Christian, Andreas Kremer, Angela Serra, Dario Greco

**Affiliations:** 1 Faculty of Medicine and Health Technology, Tampere University, Tampere, Finland; 2 BioMediTech Institute, Tampere University, Tampere, Finland; 3 ITTM S.A. - Information Technology for Translational Medicine, Esch-sur-Alzette, Luxembourg; 4 Institute of Biotechnology, University of Helsinki, Helsinki, Finland

**Keywords:** Psoriasis, Atopic dermatitis

## Abstract

We present manually curated transcriptomics data of psoriasis and atopic dermatitis patients retrieved from the NCBI Gene Expression Omnibus and EBI ArrayExpress repositories. We collected 39 transcriptomics datasets, deriving from DNA microarrays and RNA-Sequencing technologies, for a total of 1677 samples. We provide quality-checked, homogenised and preprocessed gene expression matrices and their corresponding metadata tables along with the estimated surrogate variables. These data represent a ready-made valuable source of knowledge for translational researchers in the dermatology field.

## Abstract (summary)

**Table:** 

Measurement(s)	transcriptomic data • Psoriasiform dermatitis • atopic dermatitis
Technology Type(s)	digital curation
Factor Type(s)	dermatitis type • technology type
Sample Characteristic - Organism	Homo sapiens

Machine-accessible metadata file describing the reported data: 10.6084/m9.figshare.12967916

## Background & Summary

Psoriasis (PSO) and Atopic dermatitis (AD) are among the most common inflammatory skin disorders associated with immunologic impairment. While the first signs of AD tend to appear in the early childhood, the manifestation of PSO is most common during the third decade of life^1^. Both the diseases have a substantial negative impact on the quality of life of affected patients. Although a number of therapeutic approaches have been developed in the last two decades to mitigate PSO and AD symptoms, their pathophysiology is still not completely understood^2,3^. AD is believed to be driven by epidermal barrier disruption, activation of specific T-cell subsets, and dysbiosis of the commensal skin microbiome^2^ while psoriatic inflammation is sustained by uncontrolled responses of the innate and adaptive cutaneous immune system, which lead to intense keratinocyte proliferation and dysfunctional differentiation^4^.

Transcriptomics technologies, such as DNA microarray and RNA Sequencing (RNA-Seq), have been used to characterise the molecular alterations of human diseases^5^, including PSO and AD. To date, only marginal efforts have been carried out in order to collect, quality-check and harmonize PSO- and AD-related transcriptomics data in order to make them easily reusable by the research community. Therefore, the motivation behind this study was to create a source of ready-to-use data of gene expression profiles of PSO and AD patients derived from both DNA microarray and RNA-Seq publicly available datasets.

The preprocessed and harmonized microarray data provided in this study were collected from the NCBI Gene Expression Omnibus (GEO) and EBI ArrayExpress public repositories, while the RNA-Seq datasets were retrieved from the European Nucleotide Archive (ENA). Overall, 26 microarrays datasets were collected, for a total of 991 samples, 632 of which from patients affected by psoriasis and 70 by atopic dermatitis. Some of the microarray datasets contain samples collected from patients affected by other skin diseases such as psoriatic arthritis, psoriasis sebaceous hyperplasia, palmoplantar pustulosis, lichen planus and discoid lupus. These datasets were generated with commercially available Affymetrix and Agilent platforms. All of the analytical steps performed in this work were carried out through the use of the eUtopia software^6^. We also retrieved 13 RNA-Seq datasets, for a total of 686 samples, 392 of which from patients affected by psoriasis and 94 by atopic dermatitis. RNA-seq data were mostly produced through Illumina platforms, while a minority of datasets were produced through other platforms. All the datasets underwent meta-data curation and harmonisation, data quality check and preprocessing with standardised procedures. The curation and harmonisation of the meta-data consisted in the definition and usage of a common data model for all of the collected datasets. The data models, to which the raw meta-data were mapped to, are reported in the data dictionary files (enclosed with the preprocessed data). The data dictionary describes all the variables reported in the final metadata tables. For each variable, the description, type and allowed values are reported. At the same time, this work is aimed at homogenising the preprocessing procedures in order to improve the comparability of the gene expression data across different studies and platforms. Therefore, in this work we provide meta-data tables, along with the inferred surrogate batch variables, as well as the preprocessed gene expression estimates.

Our analysis significantly increases the FAIRness^7^ of publicly available PSO and AD transcriptomics data and represents a valuable “ready-to-use” resource available to the scientific community.

## Methods

### Microarray data

#### Data collection and homogenization

Transcriptomics data generated by DNA microarrays of psoriasis and atopic dermatitis patients were retrieved from NCBI GEO^8^ (GEO - https://www.ncbi.nlm.nih.gov/geo/) and EBI ArrayExpress (https://www.ebi.ac.uk/arrayexpress/) repositories by using the R packages GEOquery^9^ and ArrayExpress^10,11^, respectively. For each dataset, a table specifying the disease (psoriasis/atopic dermatitis) and the origin of biopsy (lesional/non-lesional sample) in addition to other phenotypic information was also retrieved. Since the phenotypic information was heterogeneous across the datasets, rigorous harmonization procedure was performed. The GEO and Array Express identifiers of the retrieved datasets are reported in Tables 1–3.

**Table 1:** DNA microarray and RNA-Sequencing datasets of Atopic Dermatitis samples.

Atopic Dermatitis				
GEO dataset	# of included samples	PMID	Technology	Platform
GSE16161	16	20004782	Microarray	GPL570
GSE32924	28	21388663	Microarray	GPL570
GSE120721	50	25567045	Microarray	GPL570
GSE65832	40	25840722	RNA-Seq	GPL10999

**Table 2:** DNA microarray and RNA-Sequencing datasets of Psoriasis and Atopic Dermatitis samples.

Atopic Dermatitis and Psoriasis				
Dataset ID	# of included samples	PMID	Technology	Platform
GSE75890	27	26841714	Microarray	GPL17692
GSE121212	147	30641038	RNA-Seq	GPL16791

**Table 3:** DNA microarray and RNA-Sequencing datasets of Psoriasis samples.

Psoriasis				
Dataset ID	# of included samples	PMID	Technology	Platform
E-MTAB-3201	19	26086874	Microarray	GPL571
GSE2737	8	16283139	Microarray	GPL91
GSE6710	25	16858420	Microarray	GPL96
GSE13355	173	19169254	Microarray	GPL570
GSE14905	75	18648529	Microarray	GPL570
GSE30999	151	22763790	Microarray	GPL570
GSE34248	24	23308107	Microarray	GPL570
GSE41662	46	23308107	Microarray	GPL570
GSE50790	8	22479649	Microarray	GPL570
GSE52471	38	23771123	Microarray	GPL571
GSE58121	18	25058585	Microarray	GPL14550
GSE61281	52	25243786	Microarray	GPL6480
GSE67853	24	26763436	Microarray	GPL570
GSE68923	5	28570274	Microarray	GPL13607
GSE68924	5	28570274	Microarray	GPL13607
GSE68937	6	28570274	Microarray	GPL13607
GSE68939	5	28570274	Microarray	GPL13607
GSE78097	31	27185339	Microarray	GPL570
GSE80047	50	27152848	Microarray	GPL13158
GSE82140	8	27312025	Microarray	GPL17692
GSE83582	93	27448749	Microarray	GPL19983
GSE106087	6	Unpublished	Microarray	GPL15207
GSE41745	6	21850022	RNA-Seq	GPL10999
GSE47944	84	24909886	RNA-Seq	GPL11154
GSE54456	174	24441097	RNA-Seq	GPL9052
GSE63979	42	5723451	RNA-Seq	GPL9052
GSE67785	28	26251673	RNA-Seq	GPL10999
GSE74697	52	27793094	RNA-Seq	GPL16791
GSE83645	25	29031600	RNA-Seq	GPL10999
GSE107871	24	29273799	RNA-Seq	GPL10999
GSE117405	28	30054515	RNA-Seq	GPL11154
GSE123785	19	31539532	RNA-Seq	GPL18573
GSE123786	16	31539532	RNA-Seq	GPL11154

#### Data quality check

The retrieved datasets were thoroughly quality checked. In particular, each sample was evaluated by visual inspection of the array pseudo-images, quality check reports and density plots of probe intensities by using the eUTOPIA software^6^. Further, outlier detection step, based on the sample distributions, was performed within each dataset by using *ad hoc* R scripts (see Code Availability section).

**Fig. 1:**
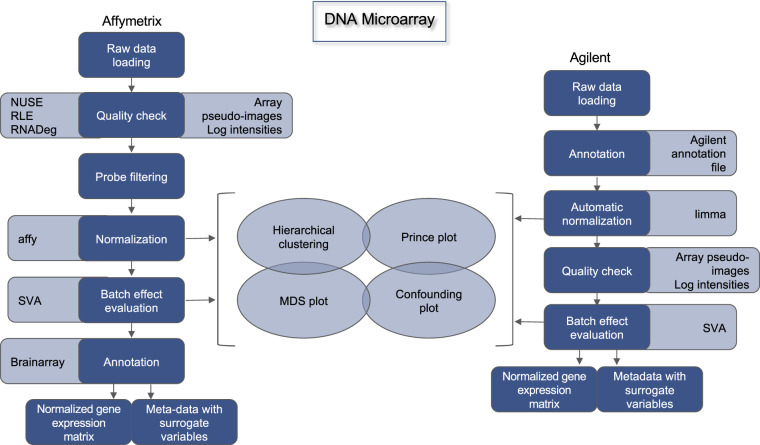
DNA microarray data preprocessing pipeline.

Moreover, for the Affymetrix datasets, outlier samples were detected by computing the Normalized Unscaled Standard Error (NUSE)^12^ and the Relative Log Expression (RLE)^12^ from the affyPLM v1.64.0 R package, and the RNA degradation curves (RNADeg)^13^ from the affy v1.64.0 R package (Fig. 1).

The distributions of the values of these three metrics were investigated by means of boxplots and the sample outlierness was evaluated for each measure based on the data distribution. Eventually, a concordance outlierness score was computed across the three metrics. In particular, a sample was removed from the analysis if considered an outlier in at least two out of three metrics, one of them being the RNA degradation curve.

#### Normalization

Data normalization was performed by using the eUTOPIA software. Affymetrixbased studies were normalized by using the justRMA from the R affy v1.66.0 package^14^. Agilent-based studies were quantile normalized with the *normalizeQuantiles* function from the limma v3.44.3 package^15^.

#### Surrogate variable analysis

In order to investigate the effect of unknown batches that might mask biological variability, Surrogate Variable Analysis (SVA) was performed with the eUtopia software, which implements the sva R package^16^. The analysis was performed by using origin of biopsy or diagnosis as variable of interest. The other biological variables (if present and if not confounded with the variable of interest) were used as covariates^6^. The estimated surrogate variables for each dataset are included in the meta-data tables.

#### Probe annotation

Custom annotation files (CDF files) were downloaded from Brainarray (http://brainarray.mbni.med.umich.edu/Brainarray/Database/CustomCDF/CDF\_download.asp) for Affymetrix-based microarrays. The latest version of Agilent probe annotation was retrieved from the Agilent website (https: //earray.chem.agilent.com/earray/). The probesets were mapped to the Ensembl gene IDs and the expression matrix was aggregated by computing the median of the expression of the Agilent probes mapping to the same Ensembl transcript ID. The entire DNA microarray data preprocessing is depicted in Fig. 1.

### RNA Sequencing data

#### Data collection and homogenization

Raw files in “*.fastq*” format were retrieved from the European Nucleotide Archive (ENA). Along with the raw data files, the metadata tables reporting the samplewise clinical features for each dataset were also collected. As for the DNA microarray data, the metadata tables of RNASeq data were carefully harmonized to improve the across-datasets comparability. Phenotypic information for each dataset is reported along with the gene expression tables. GEO and ENA identifiers of the retrieved datasets are reported in Tables 1–3.

#### Quality control

All the RNA-Seq datasets underwent quality check through the use of FastQC v0.11.7 (https://www.bioinformatics.babraham.ac.uk/projects/fastq c/). Reads were trimmed for low-quality ends in addition to adapters removal by TrimGalore v0.4.4_dev (http://www.bioinformatics.babraham.ac.uk/ projects/trim_galore/). In particular, the reads were trimmed if the Phred score was lower than 20 and discarded if the number of undetected nucleotides was greater than 50. The trimmed and adapter-clipped raw files were further quality checked with FastQC v0.11.7.

#### Read alignment

RNA Sequencing reads were then aligned against the human reference genome assembly GRCh38. The alignment was performed through the use of the HISAT2 algorithm^17,18^ using the genome indexes built for usage with HISAT2 (retrieved from https://ccb.jhu.edu/software/hisat2/manual.shtml).

Conversions between*.sam* and*.bam* file formats, sorting and extraction of uniquely mapped reads were performed through the use of samtools version 1.8-27-g0896262^19^.

#### Read counts extraction

Transcript abundance was computed by using the *featurecounts* function from the Rsubread v2.2.3 R package^20^. To accomplish this task, the Gencode version 31 annotation was downloaded from https://www.gencodegenes.org, and then utilized for read counts extraction.

#### Low counts filtering

In order to filter out the transcripts with low expression levels in all the samples of each dataset, the proportion test strategy was used as implemented in the function *filtered.data* of the R package NOISeq v2.31.0^21^.

#### Normalization

RNASeq expression data were normalized using the upper quantile method^22^ implemented in the R package NOISeq v2.31.0.

#### Surrogate Variable Analysis

**Fig. 2:**
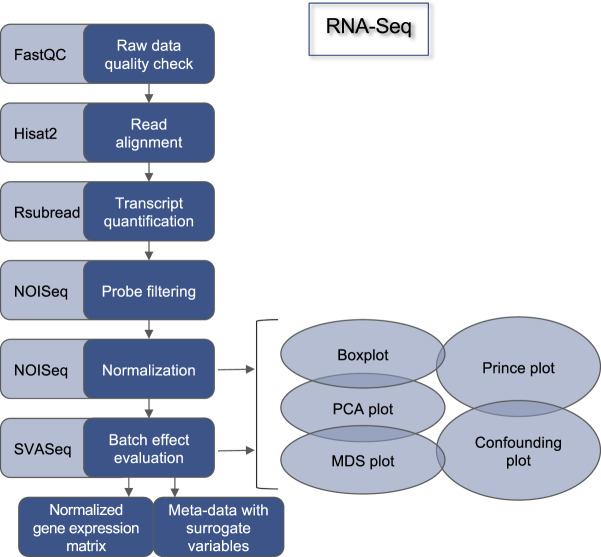
RNA-Sequencing data preprocessing pipeline.

As for the DNA microarray data, in order to identify unknown sources of technical variability, a Surrogate Variable Analysis (SVA) was performed through the use of the *svaseq* function implemented in the sva v3.36.0 R/Bioconductor package^16^. The analysis was performed by using disease state or diagnosis as variable of interest. The other biological variables (if present and if not confounded with the variable of interest) were used as covariates^6^. The estimated surrogate variables for each dataset are included in the meta-data tables, along with the gene expression tables. The entire RNA-Seq data preprocessing is depicted in Fig. 2.

## Data Records

The complete list of DNA microarray and RNA Sequencing datasets discussed in this work is reported in Tables 1–3. All the preprocessed transcriptomics data, along with harmonised meta-data, were submitted to Zenodo^23^.

## Technical Validation

DNA microarray and RNA-Seq data are linked to clinical meta-data, reporting multiple information such as *gender*, *age* or the *treatment* (including e.g. drug *dose*). Additionally, sample meta-data is recorded, such as the *tissue* type a sample was taken from, or whether the tissue derives from a *lesional* or *nonlesional* sample.

In order to ensure that the data is recorded in a consistent and well-formed way, we created data dictionaries describing each of these variables. The data dictionaries contain detailed information describing the content of a variable, the data type (numeric, categorical, text, date, etc), the allowed values of categorical data or ranges of numeric variables.

The data was validated by checking compliance with the rules encoded in the data dictionaries. Data that was found not to comply with the rules was manually curated by consulting the original data sources. In fact, a large proportion of the datasets were found not to meet the requirements encoded in the data dictionaries. For instance, big heterogeneity was found in the description of the skin status. “Involved skin”, “psoriatic skin” were reported in order to describe the “lesional” status of the skin. “Normal”, “ctrl”, “Non-involved skin of healthy individual” were used to describe the “healthy control” samples. Yet, to define the gender, “m”, “f”, “male” and “female” were used across the datasets. All of these variables were mapped to the allowed values reported in the data dictionaries to improve the comparability across the datasets.

## Usage Notes

The transcriptomics data presented in this article is an unprecedented source of preprocessed, harmonized, “ready-to-use” and FAIR datasets, made available to the scientific community. Data derived from both DNA microarray and RNASeq technologies can be exploited in order to uncover the molecular mechanisms underlying psoriasis and atopic dermatitis. Differential expression analysis can be carried for instance by the limma package^15^ for the microarray data, and the edgeR^24^, DESeq 2^25^ or NOISeq^21^ packages for RNA-Seq data, respectively. Functional analysis of differentially expressed genes can be performed by using FunMappOne^26^, the R/Bioconductor package ReactomePA^27^ or Ingenuity Pathway Analysis (Qiagen, http://www.ingenuity.com/products/ipa). The inference and analysis of co-expression networks can be performed, for instance, by using the INfORM tool^28^. Altogether, these analyses can aid the stratification of PSO and AD patients, the identification of relevant biomarkers and novel therapeutic targets.

## Data Availability

R scripts for the analysis of DNA microarray and RNA-Seq transcriptomics data are available for download at: https://github.com/Greco-Lab/psoriasis-dermatitis-analysis.
